# Complementary feeding practices among children aged 6–23 months in Gujarat, India: patterns, predictors and barriers

**DOI:** 10.3389/fpubh.2026.1713071

**Published:** 2026-02-11

**Authors:** Roshni Vakilna, Ranjit Kumar Singh, Alexandra Rutishauser, Indu Bisht, Sweta Kumari, Tanmay Mahapatra

**Affiliations:** 1 Action Against Hunger, Mumbai, India; 2 Department of Women and Child Development, Gandhinagar, Gujarat, India; 3 Action Against Hunger, London, United Kingdom; 4 Bihar Technical Support Program, Patna, Bihar, India

**Keywords:** age-appropriate minimum meal frequency, complementary feeding, infant and young child feeding practices, minimum acceptable diet, minimum dietary diversity, timely initiation of complementary feeding

## Abstract

**Background:**

Timely, adequate, and diverse complementary feeding (CF) is critical to prevent child undernutrition, a major public health concern in low- and middle-income countries, including India. Despite progress, significant disparities persist across Indian states, with Gujarat performing below the national average for several CF Indicators. This study examined infant and young child feeding (IYCF) practices, their predictors, and barriers in selected districts of Gujarat to inform targeted interventions.

**Methods:**

A cross-sectional study was conducted in four districts, with two-stage representative sampling powered for estimating district estimates. Data were collected from 1,575 and 1,583 mothers of 6–11 and 12–23 months-old children, respectively, through structured digital interviews. Socio-demographic variables, programmatic exposures, and IYCF practices were analysed using descriptive statistics and multivariable logistic regression models.

**Results:**

Mothers of 12–23 month children showed higher TICF (76.5%) and MMF (95.6%) than 6–11 months (64.8, 76.7%), while 6–11 months demonstrated better MDD (50.2% vs. 42.7%) and MAD (46.5% vs. 41.9%) practices. IYCF practices were significantly better among mothers aged 25–34 years, from non-marginalized groups, with at least primary education, and from wealthier households. Maternal employment, participation in CF-day, receipt of Balshakti (take-home ration), FLW visit, and CF-counselling were positively associated with improved IYCF practices.

**Conclusion:**

Socio-culturally sensitive and context-specific interventions are vital to improve IYCF practices. Tailored counselling and sustained FLW engagement can bridge awareness gaps, correct misperceptions, and enhance mothers’ understanding of infants’ nutritional needs. Strengthening community-based platforms and ensuring targeted outreach among marginalized groups can promote healthier feeding practices and reduce child undernutrition in Gujarat.

## Introduction

Undernutrition remains a major global public health concern affecting people of all ages. Infants and children are particularly vulnerable to undernutrition, more so in the developing world. According to the World Health Organization (WHO) (1), nearly half of the deaths among children under 5 years of age are linked to undernutrition, occurring mainly in low and middle-income countries. The physical and mental development of an infant depends critically on timely and adequate nutrition, prerequisites to enable the child to attain its full growth potential. These nutritional requirements of a normal baby are usually met by the breast milk alone until the age of 6 months (2, 3). Post that age, the needs for nutrients and energy go beyond those provided through breast milk only, raising the need for complementary feeding (CF) to meet the bodily requirements (3, 4). Moreover, this is the developmental stage for having infants adapt to healthy foods to develop long-term dietary habits (3, 5–7).

Premature cessation or less frequent breastfeeding contributes to malnutrition of young infants (5). Any delay in the timely initiation of complementary feeding (TICF) beyond 6 months may further hamper growth and development. Lack of appropriate breastfeeding and CF practices is identified as the main cause of undernutrition, leading to an estimated 148 million under-5 children being stunted (too short for age) and 45 million to be wasted (too thin for height) in 2022 globally (1, 8, 9). WHO also recommends that all infants should start receiving CF in addition to breast milk at 6 months of age (10). During the age of 6–8 months, infants should be fed complementary foods 2–3 times/day. The frequency then needs to increase to 3–4 times/day from 9 months of age till 24 months (10). Moreover, the nutritive quality of food plays the most crucial role in a child’s growth before the age of 2 years. Evidence suggests that complementary foods are often provided to children with inadequate frequency, quantity, or quality, owing to issues pertaining to awareness, perception, and availability (3, 6, 7, 11–14). Thus, children remain at a higher risk of developing undernutrition during the period of CF (15). Lack of dietary diversity is another major predictor of childhood stunting and underweight (12).

It has been well documented that nutritional interventions delivered through community engagement can improve maternal and child health, reducing mortality and accelerating progress in achieving the targets set for health outcomes (16–19). Improving maternal nutrition during pregnancy and childhood nutrition during infancy through focused interventions is critical for alleviating the burden of undernutrition (11). In 1975, the Government of India (GoI) launched the Integrated Child Development Scheme (ICDS) (20) to provide nutritional support to the children, and later introduced the Mid-Day Meal Scheme, the National Food Security Act, and the Zero Hunger Program. The nutritional indicators have consequently improved in India, corroborating betterment in socio-economic factors, food security, and health infrastructure, but the under-5 dietary outcomes still need further attention. As per the National Family Health Survey (NFHS)-5 report (2019–2021), India has persistently high levels of child undernutrition, with 36% of under-5 children being stunted, 19% being wasted, and 32% being underweight (21).

While national surveys such as the National Family Health Survey (NFHS) and the Comprehensive National Nutrition Survey (CNNS) provide robust population-level estimates of infant and young child feeding indicators, their design inherently limits the ability to capture district-specific variations, local determinants, and programmatic bottlenecks influencing feeding practices. These surveys primarily focus on outcome prevalence and broad correlates, with limited exploration of contextual, behavioral, and health-system related factors that shape IYCN practices at community and service-delivery levels. As a result, actionable evidence required to inform the design, adaptation, and implementation of district-level nutrition interventions remains insufficient, particularly in high-burden settings with substantial intra-state heterogeneity.

Given the persistent burden of child undernutrition and suboptimal IYCN practices in India and Gujarat, there is a critical need for granular, context-specific evidence that goes beyond national estimates to examine the predictors, barriers, and systemic influences on feeding behaviors during the complementary feeding period. Baseline assessments, along with qualitative inquiries conducted prior to the implementation of multi-component nutrition interventions, are essential to identify local gaps in maternal knowledge, service utilization, and frontline worker engagement, and to generate a reference framework for measuring program impact. The present study was therefore undertaken to provide district-level evidence on IYCF practices among mothers of children aged 6 to 23 months, addressing a key evidence gap not sufficiently captured by large-scale national surveys and directly informing the design and evaluation of targeted nutrition interventions.

In the Indian state of Gujarat, among under-5 children, the prevalence of undernutrition is higher than the national average, with 39% stunted, 25% wasted, and 40% underweight (22). Substantial improvements are required in the state for the nutritional indicators to achieve the global 2030 targets (23). There exists a more than 10% gap between the target and projected child wasting prevalence for Gujarat (23). The Government of Gujarat (GoG) had taken several measures to address the challenge, including the launch of the Gujarat State Nutrition Mission in 2012, with an integrated and holistic approach focusing on both preventive and curative aspects, through strengthening existing nutrition programs, but there remained several pockets with much worse conditions of nutritional indicators than the rest of the state.

To contribute to the GoG’s ongoing efforts, a project named “VRUDDHI” was planned to work closely with the GoG to increase the demand for nutrition services in communities and support the government, Front-Line Workers (FLWs), and other functionaries in providing quality health and nutrition services. This intervention was specifically designed to accelerate progress towards nutrition outcomes at the district level, which could later be advocated for scale-up at the state or national level. The project aimed to focus on four districts of Gujarat—Bhavnagar, Sabarkantha, Amreli, and Aravalli, having a high burden of stunting, low coverage of quality nutrition services, and limited knowledge of good nutrition behaviors within communities. The present study in 2021 focused on the assessment of the status of the infant and young child feeding (IYCF) practices among mothers of children aged 6–23 months, their predictors, and barriers before the actual implementation of the project initiatives to create a baseline of nutritional outcomes in the specified geography. It also explored the socio-demographic and systemic influences on IYCF practices in the first 2 years of life.

## Methods

### Study population and setting

The current analysis used data from a household survey conducted in 2021, using a representative sample of mothers of children aged 6–23 months, from four selected districts (Bhavnagar, Sabarkantha, Amreli, and Aravali) of Gujarat.

### Sample size estimation

Using the sample size calculation formula for binomial proportions [P*Q*N/(N*0.052) ÷ (1.962 + P*Q)]; where, *N* = size of the eligible population, *P* = most conservative coverage/burden (to have the most optimum sample size required for robust estimates) of 0.5, *Q* = 1−*P*, 1.96 = *z*-score for the 95% confidence interval (CI). Deciding ±5% as the width of 95% CI; assuming an *α*-error of 0.05, *β*-error of 0.2 and absolute precision of 5%, 384 samples were required to be recruited for district level estimates. Accounting for ~5% sample loss, 400 eligible subjects in each of the four districts were planned to be invited to participate in the study from each of the two groups, i.e., mothers of children aged 6–11 months (Group-A) and 12–23 months (Group-B). Thus, the final target sample size was 400*4 = 1,600 for each group. After the data cleaning and management, the final study sample used for the analysis was 1,575 mothers of children aged 6–11 months and 1,583 mothers of children aged 12–23 months, representative of the four districts.

### Study design and sampling strategy

The study employed a cross-sectional design and adopted a two-stage sampling strategy. Proportional random sampling at the Anganwadi Centre (AWC) level in the first stage was followed by a systematic sampling with a random start for the selection of the households with eligible mothers having children aged 6–23 months. Four hundred AWCs were randomly selected from each district (covering all blocks), ensuring proportional representation of the AWC populations for both tribal and rural areas. One interview per group (Group-A: mothers of children aged 6–11 months and Group-B: mothers of children aged 12–23 months) was conducted at each selected AWC catchment area.

### Data collection

The pre-validated survey questionnaire captured a wide range of information from mothers of children aged 6–23 months, related to socio-economic characteristics, IYCF practices, FLW, and ICDS services. All interviews were conducted during August–September 2021, face-to-face by trained data collectors in the local language using handheld tablet-computers. Before the interview, informed consents were obtained from respondents. Consent was documented electronically using the Survey CTO data collection platform, where participants provided informed consent after the study objectives, procedures, potential risks, and benefits were explained in the local language, owing to low literacy levels. Participants indicated their consent digitally before the interview could proceed.

### Outcome measures and covariates

Five key outcome indicators related to CF practices were measured: (i) timely initiation of complementary feeding (TICF), (ii) minimum dietary diversity (MDD), (iii) minimum meal frequency (MMF), (iv) appropriate meal quantity (AMQ), and (v) minimum acceptable diet (MAD). TICF was defined as the introduction of solid/semi-solid/soft foods to the infants at six months of age. MDD was defined as children receiving complementary foods from ≥four (among seven) diverse food groups. MMF was defined by age-appropriate meal frequency/day, i.e., the infant is fed complementary foods 2 times/day for 6–8 months and 3 times/day between 9–11 and 12–23 months. AMQ constituted > = 200 mL meal quantity for 6–8 months and > = 300 mL meal quantity for 9–11 months old infant. Minimum acceptable diet is defined as receiving both MDD and MMF (24, 25).

The socio-economic factors captured in the study were mother’s age, religion, caste, mother’s highest educational level, employment status, and household wealth index tertile (determined based on a context-specific asset list). Exposure to programmatic services captured in the study included: mother’s participation in the community event called “Complementary Feeding (CF) Day”, receiving Balshakti from the Anganwadi centre, FLW visit, and services. Balshakti is a Take-Home Ration (THR) that is part of the Supplementary Nutrition Programme (SNP) of Integrated Child Development Services (ICDS), providing 500 calories and 12–15 g of protein/day. The FLW Counselling Index was also created to quantify the extent of appropriate counselling provided to the mothers on TICF, MMF, AMQ, MDD, and handwashing. Each of these counselling items, if conducted, was given a score of 1 (else 0), summed up, and the average score was categorized into “poor,” “average,” and “good” based on tertile boundaries.

### Statistical analysis

Descriptive analysis (frequencies, proportions, and corresponding 95%-CIs) was used to determine the distribution of various parameters in the study population. Multivariable Logistic Regression was employed to identify [adjusted Odds Ratios (aOR) and corresponding *p*-values] enablers and barriers to complementary feeding practices among children aged 6–23 months after adjusting for mother’s age, religion, caste, education, employment status, and wealth tertile. All analyses were conducted using SAS version 9.4.

In stratified and multivariable analyses, certain exposure-outcome combinations resulted in sparse cell counts, particularly for age-specific programmatic variables and low-prevalence outcomes such as age-appropriate meal quantity. Where sparse data led to unstable estimates or model non-convergence, results were not reported and are indicated as “–” in the tables. These instances primarily reflect either the age-inapplicability of specific variables or insufficient observations within strata to support reliable estimation.

### Ethical approval

The study protocol and procedures were reviewed and approved by the Ashirwad Ethics Committee, Ashirwad Hospital and Research Centre, Ulhasnagar, India. Verbal informed consent was collected from each agreeing participant before the interview, after explaining the details of the study in a language that they could understand.

## Results

### Distribution of the study population by select characteristics

A total of 1,575 mothers of children aged 6–11 months and 1,583 mothers of children aged 12–23 months participated in the study. Across both groups, most mothers (55.7% in 6–11 months and 57.9% in 12–23 months) were aged 25–34 years, nearly 95% were Hindu, and one-third belonged to marginalized communities. About one-fourth of the mothers (21.7% in 6–11 months and 23.9% in 12–23 months) had no formal education, and over 80% were not engaged in paid work.

Approximately one-third of the mothers with children aged 6–11 months participated in the CF-day in the last 3 months. More than 90% in each group reported receiving Balshakti from AWCs, while 84% were visited by frontline workers within 6 months postpartum. However, only 30% received good-quality counselling on all key CF components as per the FLW Counselling Index (Table 1).

**Table 1:** Distribution of socio-demographic characteristics and programmatic exposures/ support among study participants (mothers of children aged 6–11 and 12–23 months).

Description	Categories	6–11 m (*N* = 1,575)	12–23 m (*N* = 1,583)
		% (95% CI)	% (95% CI)
Mother’s age	<25 years	39.6 (37.1–42.0)	35.4 (33.1–37.8)
	25–34 years	55.7 (53.2–58.1)	57.9 (55.4–60.3)
	>34 years	4.8 (3.7–5.8)	6.7 (5.5–7.9)
Mother’s religion	Hindu	94.8 (93.7–95.9)	95.3 (94.3–96.4)
	Non-Hindu	5.2 (4.1–6.3)	4.7 (3.6–5.7)
Mother’s caste	Marginalized	34.5 (32.1–36.8)	36.1 (33.8–38.5)
	Non-marginalized	65.5 (63.2–67.9)	63.9 (61.5–66.2)
Mother’s education	No formal education	21.7 (19.6–23.7)	23.9 (21.8–26.1)
	Up to 8th	36.8 (34.4–39.2)	37.9 (35.5–40.3)
	Above 8th	41.6 (39.2–44.0)	38.2 (35.8–40.6)
Mother’s wealth tertile	Lower tertile	33.3 (30.9–35.6)	33.3 (31.0–35.6)
	Middle tertile	33.5 (31.1–35.8)	33.4 (31.0–35.7)
	Upper tertile	33.3 (30.9–35.6)	33.4 (31.0–35.7)
Mother’s employment status	Working	14.5 (12.7–16.2)	16.6 (14.7–18.4)
	Not working	85.5 (83.8–87.3)	83.5 (81.6–85.3)
Mother participated in CF-day in last 3 months		33.0 (30.6–35.3)	–
Received Balshakti from Anganwadi centre		91.6 (90.3–93.0)	92.9 (91.7–94.2)
Visited by FLW during 6 months after delivery		84.3 (82.5–86.1)	–
Received any advice on complementary feeding from FLW		72.2 (70.0–74.4)	–
Received appropriate counselling from FLW on initiation of complementary feeding		61.6 (59.2–64.0)	–
Received appropriate counselling from FLW on minimum meal frequency		54.3 (51.8–56.8)	–
Received appropriate counselling from FLW on age-appropriate meal quantity		9.8 (8.3–11.3)	–
Received appropriate counselling from FLW on minimum dietary diversity		41.3 (38.8–43.7)	–
FLW counselling index	Poor	31.5 (29.2–33.8)	–
	Average	38.0 (35.6–40.4)	–
	Good	30.5 (28.2–32.8)	–

*N* = group-specific total number of participants.

95% CI = 95% confidence interval.

### Complementary feeding practices

**Figure 1:**
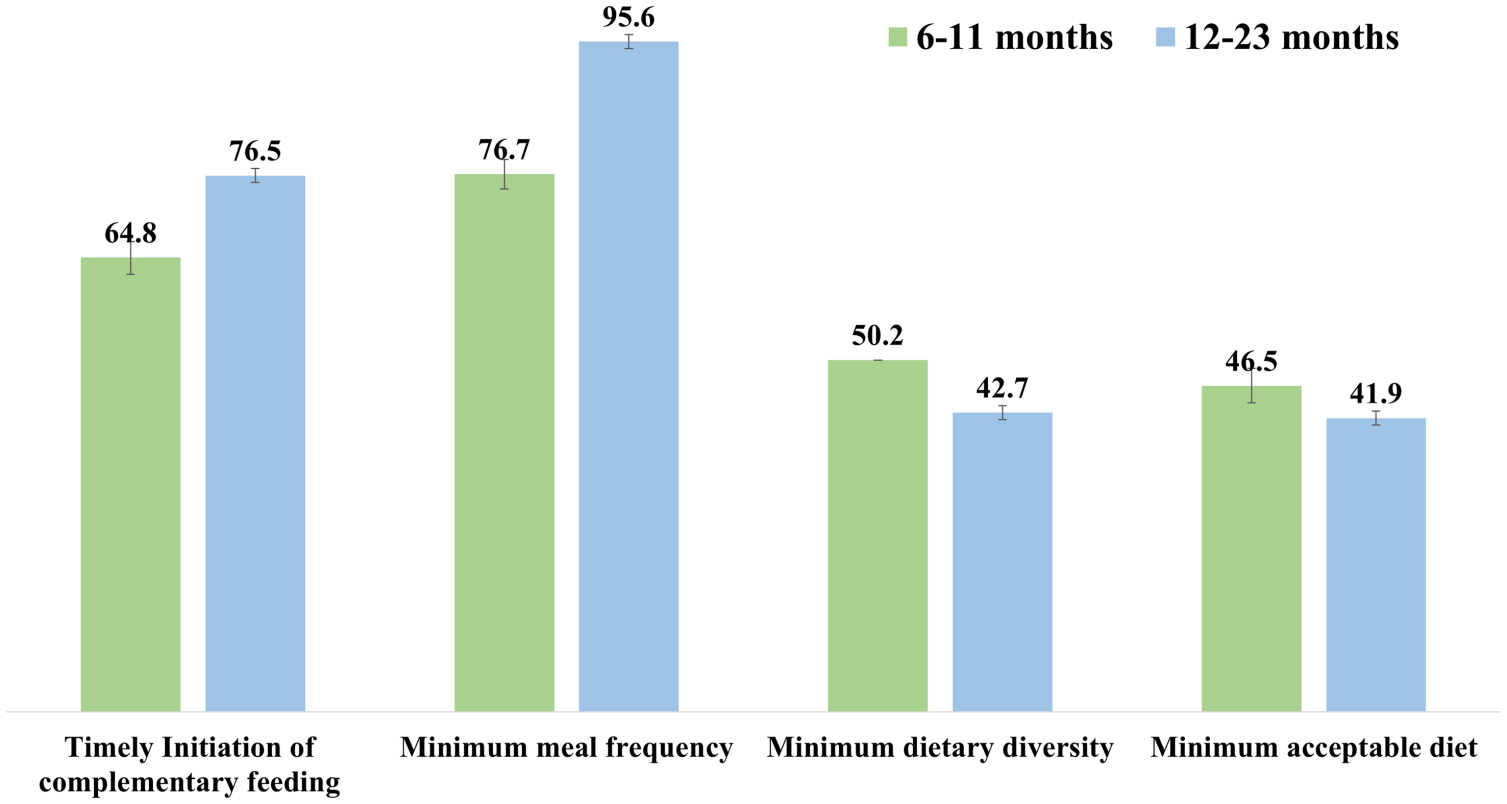
Distribution of infant and young child feeding practices among children aged 6–23 months.

A total of 64.8% mothers of children aged 6–11 months and 76.5% mothers of children aged 12–23 months initiated CF of their children in a timely manner. Age-appropriate MMF was adhered to by 76.7% Group-A and 95.6% Group-B mothers. Only 8.4% mothers (of children aged 6–11 months) provided their children with AMQ. Half (50.2%) of the 6–11 months old and less than half (42.7%) of the 12–23 months old babies had MDD. Furthermore, less than half of the mothers (46.5% in Group-A and 41.9% in Group-B) provided MAD to their children (Figure 1).

**Figure 2:**
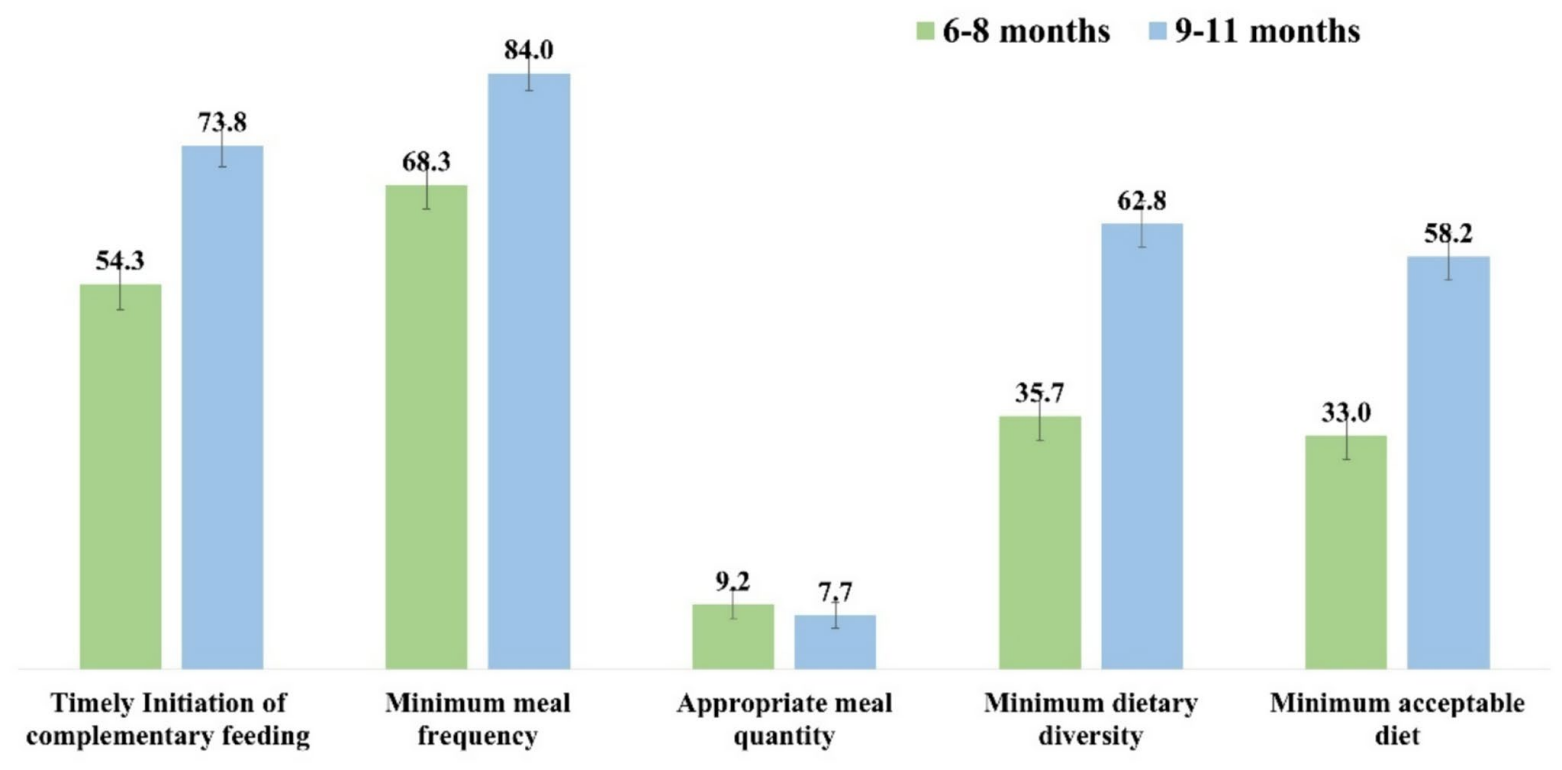
Distribution of complementary feeding practices among children aged 6–8 months and 9–11 months with 95% confidence interval.

Younger infants (6–8 months) had markedly lower adherence to TICF, MDD, and MAD compared with older infants (9–11 months). For instance, TICF (54.3% vs. 73.8%), MDD (35.7% vs. 62.8%), and MAD (33.0% vs. 58.2%) improved with child age, though AMQ remained consistently low across both groups (see Figure 2).

### Distribution of complementary feeding practices across socio-demographic characteristics and programmatic exposures

Among mothers of children 6–11 months old, those aged 25–34 years had better CF practices, compared to younger (<25 years) and older mothers (>34 years), particularly for MMF (78.2%). A lower proportion of mothers from marginalized communities practiced TICF (57.6%), MDD (41.6%), and MAD (38.3%) as compared to non-marginalized. CF practices were the lowest among mothers with no formal education and in the lower wealth tertile for most outcome indicators. Working mothers appeared to be doing better with MDD (58.3%) and MAD (53.1%), while non-working mothers showed better adherence to TICF (66.4%) (Table 2).

**Table 2:** Distribution of complementary feeding practices for children aged 6–11 months across socio-demographic characteristics and programmatic exposures.

Description	Categories	Timely initiation of complementary feeding		Minimum meal frequency		Age-appropriate meal quantity		Minimum dietary diversity		Minimum acceptable diet	
		*n*	% (95% CI)	*n*	% (95% CI)	*n*	% (95% CI)	*n*	% (95% CI)	*n*	% (95% CI)
Mother’s age	<25 years	395	63.4 (59.6–67.2)	464	74.5 (71.1–77.9)	49	7.9 (5.8–10.0)	308	49.4 (45.5–53.4)	284	45.6 (41.7–49.5)
	25–34 years	576	65.7 (62.5–68.8)	686	78.2 (75.5–81.0)	79	9.0 (7.1–10.9)	441	50.3 (47.0–53.6)	410	46.8 (43.4–50.1)
	>34 years	49	65.3 (54.6–76.1)	58	77.3 (67.9–86.8)	4	5.3 (0.2–10.4)	42	56.0 (44.8–67.3)	38	50.7 (39.3–62.0)
Mother’s religion	Hindu	967	64.8 (62.3–67.2)	1,143	76.6 (74.4–78.7)	123	8.2 (6.8–9.6)	741	49.6 (47.1–52.2)	687	46.0 (43.5–48.6)
	Non-Hindu	53	64.6 (54.3–75.0)	65	79.3 (70.5–88.1)	9	11.0 (4.2–17.8)	50	61.0 (50.4–71.6)	45	54.9 (44.1–65.7)
Mother’s caste	Marginalized	313	57.6 (53.5–61.8)	398	73.3 (69.6–77.0)	41	7.6 (5.3–9.8)	226	41.6 (37.5–45.8)	208	38.3 (34.2–42.4)
	Non-marginalized	707	68.5 (65.7–71.3)	810	78.5 (76.0–81.0)	91	8.8 (7.1–10.6)	565	54.8 (51.7–57.8)	524	50.8 (47.7–53.8)
Mother’s education	No formal education	196	57.5 (52.2–62.7)	239	70.1 (65.2–75.0)	20	5.9 (3.4–8.4)	139	40.8 (35.5–46.0)	124	36.4 (31.3–41.5)
	Up to 8th	391	67.5 (63.7–71.4)	448	77.4 (74.0–80.8)	51	8.8 (6.5–11.1)	315	54.4 (50.3–58.5)	291	50.3 (46.2–54.3)
	Above 8th	433	66.1 (62.5–69.7)	521	79.5 (76.5–82.6)	61	9.3 (7.1–11.5)	337	51.5 (47.6–55.3)	317	48.4 (44.6–52.2)
Mother’s wealth tertile	Lower tertile	305	58.2 (54.0–62.4)	374	71.4 (67.5–75.3)	33	6.3 (4.2–8.4)	207	39.5 (35.3–43.7)	187	35.7 (31.6–39.8)
	Middle tertile	356	67.6 (63.6–71.6)	420	79.7 (76.3–83.1)	42	8.0 (5.7–10.3)	276	52.4 (48.1–56.6)	258	49.0 (44.7–53.2)
	Upper tertile	359	68.5 (64.5–72.5)	414	79.0 (75.5–82.5)	57	10.9 (8.2–13.6)	308	58.8 (54.6–63.0)	287	54.8 (50.5–59.0)
Mother’s employment status	Not working	894	66.4 (63.8–68.9)	1,040	77.2 (75.0–79.5)	108	8.0 (6.6–9.5)	658	48.9 (46.2–51.5)	611	45.4 (42.7–48.0)
	Working	126	55.3 (48.8–61.7)	168	73.7 (68.0–79.4)	24	10.5 (6.5–14.5)	133	58.3 (51.9–64.7)	121	53.1 (46.6–59.6)
Mother participated in CF-day in last 3 months	No	660	62.5 (59.6–65.4)	783	74.2 (71.5–76.8)	70	6.6 (5.1–8.1)	482	45.6 (42.6–48.7)	441	41.8 (38.8–44.7)
	Yes	360	69.4 (65.4–73.3)	425	81.9 (78.6–85.2)	62	12.0 (9.2–14.7)	309	59.5 (55.3–63.8)	291	56.1 (51.8–60.3)
Received Balshakti from Anganwadi centre	No	74	56.1 (47.6–64.5)	90	68.2 (60.2–76.1)	7	5.3 (1.5–9.1)	49	37.1 (28.9–45.4)	47	35.6 (27.4–43.8)
	Yes	946	65.6 (63.1–68.0)	1,118	77.5 (75.3–79.6)	125	8.7 (7.2–10.1)	742	51.4 (48.8–54.0)	685	47.5 (44.9–50.1)
Visited by FLW during 6 months after delivery	No	137	55.2 (49.1–61.4)	162	65.3 (59.4–71.3)	15	6.1 (3.1–9.0)	83	33.5 (27.6–39.4)	73	29.4 (23.8–35.1)
	Yes	883	66.5 (64.0–69.1)	1,046	78.8 (76.6–81.0)	117	8.8 (7.3–10.3)	708	53.4 (50.7–56.0)	659	49.7 (47.0–52.4)
Received any advice on complementary feeding from FLWs	No	230	52.5 (47.8–57.2)	287	65.5 (61.1–70.0)	20	4.6 (2.6–6.5)	153	34.9 (30.5–39.4)	136	31.1 (26.7–35.4)
	Yes	790	69.5 (66.8–72.2)	921	81.0 (78.7–83.3)	112	9.9 (8.1–11.6)	638	56.1 (53.2–59.0)	596	52.4 (49.5–55.3)
Received appropriate counselling on specific components of complementary feeding from FLWs	No	324	53.6 (49.6–57.5)	420	67.2 (63.8–70.7)	109	7.7 (6.3–9.1)	354	38.3 (35.1–41.4)	–	–
	Yes	696	71.8 (68.9–74.6)	788	84.7 (82.3–87.1)	23	14.9 (9.3–20.6)	437	67.2 (63.6–70.8)	–	–
FLW counselling index	Poor	256	51.6 (47.2–56.0)	322	64.9 (60.7–69.1)	25	5.0 (3.1–7.0)	170	34.3 (30.1–38.5)	152	30.7 (26.6–34.7)
	Average	401	66.9 (63.2–70.7)	472	78.8 (75.5–82.1)	48	8.0 (5.8–10.2)	301	50.3 (46.2–54.3)	277	46.2 (42.3–50.2)
	Good	363	75.6 (71.8–79.5)	414	86.3 (83.2–89.3)	59	12.3 (9.4–15.2)	320	66.7 (62.4–70.9)	303	63.1 (58.8–67.5)

“–"indicates analysis without valid results, owing to sparse data problems.

*n* = Starum-specific number of participants. 95% CI = 95% confidence interval.

Participation in CF-day and receiving Balshakti from AWCs were also found to influence the CF practices among Group-A mothers. Among those who participated in CF-day, 69.4% practiced TICF, MMF ensured by 81.9%, AMQ 12%, MDD 59.5 and 56.1% met the MAD criteria as compared to CF-day non-participants. Similarly, those who received Balshakti had better CF practices across all outcome indicators as compared to those who did not. A total of 66.5% of mothers, who were visited by FLWs for the first 6 months post-delivery, did TICF, as compared to 55.2% among non-visited. Likewise, a 13–20% point higher MMF, MDD, and MAD were observed among mothers who were visited by FLWs as compared to those who were not visited. Mothers who received any advice from the FLW on CF had better practices for all the outcomes as compared to non-recipient (69.5% vs. 52.5% for TICF; 81% vs. 65.5% for MMF; 9.9% vs. 4.6% for AMQ; 56.1% vs. 34.9% for MDD; and 52.4% vs. 31.1% for MAD). Among those who received specific component-wise counselling for CF, 71.8% (vs. 53.6% among not counselled) practiced TICF, 84.7% (vs. 67.2%) practiced MMF, 14.9% (vs. 7.7%) provided AMQ, and 67.2% (vs. 38.3%) ensured MDD for their babies. Based on the FLW counselling index, the more the counselling was provided to the mothers of children aged 6–8 months, the better were their CF practices (75.6% for TICF; 86.3% for MMF; 12.3% for AMQ; 66.7% for MDD; and 63.1% for MDD among best counselled) compared to less counselled (Table 2).

For 12–23 months old children, mothers aged 25–34 years had better adherence to TICF (79.3%). Compared to Hindu, non-Hindu mothers had better TICF (81.1%), MDD (56.8%), and MAD (56.8%). Non-marginalized mothers showed better MDD (45.3% vs. 38.1%) and MAD (44.1% vs. 37.9%) as opposed to marginalized communities. Non-working mothers had a higher percentage of TICF (78.6%) compared to working mothers (66%), but working mothers practiced better in terms of MDD (51.9% vs. 40.9%) and MAD (51.5% vs. 40%). Moreover, mothers who received Balshakti from Anganwadi centres did better for all outcome indicators (Table 3).

**Table 3:** Infant and young child feeding practices for children aged 12–23 months across socio-demographic characteristics and programmatic exposures.

Description	Categories	Timely initiation of complementary feeding		Minimum meal frequency		Minimum dietary diversity		Minimum acceptable diet	
		*n*	% (95% CI)	*n*	% (95% CI)	*n*	% (95% CI)	*n*	% (95% CI)
Mother’s age	<25 years	404	72.0 (68.3–75.7)	535	95.4 (93.6–97.1)	232	41.4 (37.3–45.4)	227	40.5 (36.4–44.5)
	25–34 years	726	79.3 (76.6–81.9)	876	95.6 (94.3–97.0)	398	43.5 (40.2–46.7)	391	42.7 (39.5–45.9)
	>34 years	81	76.4 (68.3–84.5)	102	96.2 (92.6–99.9)	46	43.4 (34.0–52.8)	45	42.5 (33.0–51.9)
Mother’s religion	Hindu	1,151	76.3 (74.1–78.4)	1,442	95.6 (94.5–96.6)	634	42.0 (39.5–44.5)	621	41.2 (38.7–43.6)
	Non-Hindu	60	81.1 (72.2–90.0)	71	96.0 (91.5–100)	42	56.8 (45.5–68.1)	42	56.8 (45.5–68.1)
Mother’s caste	Marginalized	419	73.3 (69.6–76.9)	549	96.0 (94.4–97.6)	218	38.1 (34.1–42.1)	217	37.9 (34.0–41.9)
	Non-marginalized	792	78.3 (75.8–80.9)	964	95.4 (94.1–96.7)	458	45.3 (42.2–48.4)	446	44.1 (41.1–47.2)
Mother’s education	No formal education	262	69.1 (64.5–73.8)	358	94.5 (92.2–96.8)	143	37.7 (32.9–42.6)	141	37.2 (32.3–42.1)
	Up to 8th	465	77.5 (74.2–80.8)	574	95.7 (94.0–97.3)	284	47.3 (43.3–51.3)	276	46.0 (42.0–50.0)
	Above 8th	484	80.1 (77.0–83.3)	581	96.2 (94.7–97.7)	249	41.2 (37.3–45.2)	246	40.7 (36.8–44.7)
Mother’s wealth tertile	Lower tertile	357	67.7 (63.8–71.7)	490	93.0 (90.8–95.2)	170	32.3 (28.3–36.3)	167	31.7 (27.7–35.7)
	Middle tertile	434	82.2 (78.9–85.5)	507	96.0 (94.4–97.7)	234	44.3 (40.1–48.6)	230	43.6 (39.3–47.8)
	Upper tertile	420	79.6 (76.1–83.0)	516	97.7 (96.5–99.0)	272	51.5 (47.3–55.8)	266	50.4 (46.1–54.7)
Mother’s employment status	Not working	1,038	78.6 (76.4–80.8)	1,260	95.4 (94.3–96.5)	540	40.9 (38.2–43.5)	528	40.0 (37.3–42.6)
	Working	173	66.0 (60.3–71.8)	253	96.6 (94.4–98.8)	136	51.9 (45.9–58.0)	135	51.5 (45.5–57.6)
Received Balshakti from Anganwadi centre	No	76	67.9 (59.2–76.5)	105	93.8 (89.3–98.2)	45	40.2 (31.1–49.3)	44	39.3 (30.2–48.3)
	Yes	1,135	77.2 (75.0–79.3)	1,408	95.7 (94.7–96.8)	631	42.9 (40.4–45.4)	619	42.1 (39.6–44.6)

*n* = Starum-specific number of participants.

95% CI = 95% confidence interval.

Mothers with no formal education and those from the lowest wealth tertile demonstrated poorer practices for all the outcome indicators.

### Factors influencing complementary feeding practices

Multivariable Logistic Regression of CF practices by all respondent mothers of 6–23 months old children revealed that TICF was significantly better (aOR = 1.3) by mothers aged 25–34 years as compared to younger (<25 years). Higher odds (aOR = 1.4) of MDD and MAD were observed among children in non-Hindu families (vs. Hindu). Mothers from non-marginalised communities (vs. marginalized) and educated up to Class 8 (vs. uneducated) were more likely to practice TICF, provide MDD, and MAD for their children. Women belonging to wealthier households were more likely to practice better for all the IYCF practices (aOR_TICF_ = 1.4; aOR_AMQ_ = 1.5; aOR_MDD_ = 2.0; aOR_MAD_ = 2.0) as compared to women from poorer households. Mother’s employment was negatively associated with TICF (aOR = 0.7) but positively associated with MDD (aOR = 1.8) and MAD (aOR = 1.7). Mothers who had participated in CF-day in 3 months prior to the survey were more likely to have better IYCF practices. Receiving Balshakti also ensured better IYCF practices, except for MAD. Mothers who were visited by the FLWs for 6 months post-delivery and those who received specific counselling on CF, performed better for all the IYCF practices as compared to their non-visited and non-counselled counterparts, respectively (Table 4).

**Table 4:** Factors influencing infant and young child feeding practices for children aged 6–23 months.

Description	Categories	Timely Initiation of complementary feeding		Minimum meal frequency		Minimum dietary diversity		Minimum acceptable diet	
		aOR	*p* value	aOR	*p* value	aOR	*p* value	aOR	*p* value
Mother’s age (Ref: <25 years)	25–34 years	**1.3**	**0.0072**	1.2	0.0651	1.0	0.9142	1.0	0.9202
	>34 years	1.3	0.1548	1.5	0.1315	1.1	0.4265	1.1	0.4669
Mother’s religion (Ref = Hindu)	Non-Hindu	0.9	0.7136	1.0	0.9935	**1.4**	**0.0316**	**1.4**	**0.0487**
Mother’s caste (Ref = marginalised)	Non-Marginalised	**1.4**	**0.0002**	1.1	0.3808	**1.3**	**0.0003**	**1.3**	**0.0013**
Mother’s education (Ref = no formal education)	Up to 8th	**1.4**	**0.0038**	1.2	0.1565	**1.3**	**0.0053**	**1.3**	**0.0068**
	Above 8th	**1.4**	**0.0016**	1.3	0.0712	1.1	0.4907	1.1	0.3338
Mother’s wealth tertile (Ref = lowest tertile)	Middle Tertile	**1.6**	**<0.0001**	**1.5**	**0.0034**	**1.5**	**<0.0001**	**1.6**	**<0.0001**
	Highest Tertile	**1.4**	**0.0016**	**1.5**	**0.0053**	**2.0**	**<0.0001**	**2.0**	**<0.0001**
Mother’s employment status (Ref: not working)	Working	**0.7**	**<0.0001**	1.1	0.6727	**1.8**	**<0.0001**	**1.7**	**<0.0001**
Mother participated in CF-day in last 3 months (Ref: no)	Yes	**1.4**	**0.0050**	**1.6**	**0.0008**	**1.8**	**<0.0001**	**1.9**	**<0.0001**
Received Balshakti from Anganwadi centre (Ref: no)	Yes	**1.5**	**0.0039**	**1.6**	**0.0094**	**1.3**	**0.0442**	1.3	0.0892
Visited by FLW during 6 months after delivery (Ref: no)	Yes	**1.5**	**0.0045**	**1.8**	**<0.0001**	**2.1**	**<0.0001**	**2.2**	**<0.0001**
Received any advice on complementary feeding from FLWs (Ref: no)	Yes	**1.9**	**<0.0001**	**2.0**	**<0.0001**	**2.2**	**<0.0001**	**2.2**	**<0.0001**

aOR = odds ratios from multivariable logistic regressions adjusting for mother’s age, religion, caste, education, employment status and wealth tertile.

Bold-faced figures indicate statistically significant results for which *p* < 0.05.

While diving deep to understand the practices among mothers of children aged 611 and 12–23 months separately, it was revealed that participation in CF-day in last 3 months (aOR_6–11_ = 1.4), receiving Balshakti from AWC (aOR_6–11_ = 1.4; aOR_12–23_ = 1.5), FLW visit during 6 months after delivery (aOR_6–11_ = 1.5), any advice on CF from FLWs (aOR_6–11_ = 1.9), and counselling from.

FLW on specific CF practices (aOR_6–11_ = 2.1), all played a positive role in TICF. Better FLW counselling showed significantly higher odds of TICF (aOR_average_ = 1.8; aOR_good_ = 2.7). For MMF, non-marginalised communities did better for 6–11 months aged children, whereas, for 12–23 months old, richer households performed better. AMQ was provided to 6–11 months old children by mothers who participated in CF-day (aOR = 1.9) and received better counselling. MDD and MAD were better among non-marginalised, educated, working mothers and richer households for both the age-groups. In addition, mother’s participation in CF-day, Balshakti receival, FLW visits, any, specific and good CF counselling, all were positively associated with MDD and MAD for children aged 6–11 months (Table 5).

**Table 5:** Factors influencing infant and young child feeding practices for children aged 6–11 months and 12–23 months.

Description	Categories	Timely Initiation of complementary feeding				Minimum meal frequency				Age-appropriate meal quantity		Minimum dietary diversity				Minimum acceptable diet			
		6–11 m		12–23 m		6–11 m		12–23 m		6–11 m		6–11 m		12–23 m		6–11 m		12–23 m	
		aOR	*p* value	aOR	*p* value	aOR	*p* value	aOR	*p* value	aOR	*p* value	aOR	*p* value	aOR	*p* value	aOR	*p* value	aOR	*p* value
Mother’s age (Ref: <25 years)	25–34 years	1.1	0.4633	**1.4**	**0.0050**	1.2	0.1002	0.9	0.7809	1.1	0.5198	1.0	0.9784	1.0	0.9731	1.0	0.9100	1.0	0.9690
	>34 years	1.2	0.5791	1.3	0.3094	1.3	0.4010	1.1	0.8836	0.7	0.5050	1.4	0.1738	1.0	0.9123	1.3	0.2537	1.0	0.9214
Mother’s religion (Ref = Hindu)	Non-Hindu	0.8	0.4710	1.1	0.6746	1.0	0.8915	1.0	0.9483	1.3	0.5337	1.3	0.2419	1.6	0.0628	1.2	0.4615	**1.7**	**0.0385**
Mother’s caste (Ref = marginalised)	Non-Marginalised	**1.5**	**0.0002**	1.3	0.0769	**1.3**	**0.0487**	0.7	0.2265	1.1	0.7051	**1.5**	**0.0002**	1.1	0.2112	**1.5**	**0.0004**	1.1	0.3714
Mother’s education (Ref = no formal education)	Up to 8th	**1.4**	**0.0231**	**1.4**	**0.0485**	1.3	0.0727	1.0	0.9577	1.4	0.2878	**1.5**	**0.0089**	1.2	0.1896	**1.5**	**0.0065**	1.2	0.2702
	Above 8th	**1.4**	**0.0393**	**1.6**	**0.0033**	**1.6**	**0.0064**	1.0	0.8894	1.4	0.2639	1.3	0.0839	0.9	0.3418	**1.4**	**0.0306**	0.9	0.3591
Mother’s wealth tertile (Ref = lowest tertile)	Middle Tertile	**1.3**	**0.0364**	**1.9**	**<0.0001**	**1.4**	**0.0259**	**1.9**	**0.0249**	1.2	0.5157	**1.5**	**0.0024**	**1.6**	**0.0002**	**1.5**	**0.0015**	**1.6**	**0.0002**
	Highest Tertile	1.3	0.0725	**1.5**	**0.0148**	1.2	0.2021	**3.5**	**0.0005**	1.6	0.0600	**1.8**	**<0.0001**	**2.2**	**<0.0001**	**1.8**	**<0.0001**	**2.2**	**<0.0001**
Mother’s employment status (Ref: not working)	Working	**0.7**	**0.0200**	**0.6**	**0.0005**	0.9	0.6093	1.5	0.2815	1.4	0.1365	**1.8**	**0.0001**	**1.8**	**<0.0001**	**1.7**	**0.0009**	**1.8**	**<0.0001**
Mother participated in the CF-day in the last 3 months (Ref: no)	Yes	**1.4**	**0.0050**	–	–	**1.4**	**0.0205**	–	–	**1.9**	**0.0004**	**1.8**	**<0.0001**	–	–	**1.9**	**<0.0001**	–	–
Received Balshakti from Anganwadi centre (Ref: no)	Yes	**1.4**	**0.0497**	1.5	0.0553	**1.6**	**0.0270**	1.4	0.4192	1.6	0.2300	**1.7**	**0.0064**	1.0	0.8486	**1.5**	**0.0258**	**1.0**	**0.8197**
Received a visit from FLW for 6 months after delivery (Ref: no)	Yes	**1.5**	**0.0045**	–	–	**1.8**	**<0.0001**	–	–	1.4	0.2104	**2.1**	**<0.0001**	–	–	**2.2**	**<0.0001**	–	–
Received any advice on complementary feeding from FLWs (Ref: no)	Yes	**1.9**	**<0.0001**	–	–	**2.0**	**<0.0001**	–	–	**2.1**	**0.0032**	**2.2**	**<0.0001**	–	–	**2.2**	**<0.0001**	–	–
Received appropriate counselling on specific components of complementary feeding from FLWs (Ref: no)	Yes	**2.1**	**<0.0001**	–	–	**2.5**	**<0.0001**	–	–	**2.0**	**0.0048**	**3.1**	**<0.0001**	–	–	–	–	–	–
FLW counselling index (Ref: poor)	Average	**1.8**	**<0.0001**	–	–	**1.9**	**<0.0001**	–	–	1.6	0.0863	**1.8**	**<0.0001**	–	–	**1.8**	**<0.0001**	–	–
	Good	**2.7**	**<0.0001**	–	–	**3.1**	**<0.0001**	–	–	**2.4**	**0.0005**	**3.5**	**<0.0001**	–	–	**3.5**	**<0.0001**	–	–

aOR = odds ratios from multivariable logistic regressions adjusting for mother’s age, religion, caste, education, employment status and wealth tertile.

Bold-faced figures indicate statistically significant results for which *p* < 0.05.

“–"indicates analysis without valid results, owing to sparse data problems.

## Discussion

This study examined the distribution and determinants of CF practices among a representative sample of mothers of children aged 6–23 months in the selected districts of Gujarat. While a significant proportion of children, especially in wealthier families with educated, working mothers, received complementary foods at the appropriate time, quite a few were lagging behind in timely initiation, negatively affecting their nutritional outcomes (26). A higher proportion of MMF among the children aged 12–23 months, as compared to children aged 6–11 months, indicates a better adherence to feeding guidelines by the mothers as the infants grow. Misperception and lack of awareness among caregivers regarding the required frequency of feeding for the children aged 6–11 months are potential barriers (3, 13, 14). The extremely low proportion of children aged 6–11 months receiving MMF and AMQ further reiterates a big gap in awareness of the mothers regarding how much to feed the children with respect to age, raising concerns over the lack of nutrient provision to the growing children. General misconceptions are that small kids need a very small amount of only a few types of food, not very frequently, and any chance of overfeeding may be more harmful than less feeding. Another major concern of the mothers is which food will end up in indigestion and diarrhoea (3, 13, 14). These probably culminate in the lower levels of MDD and MAD for the children as observed, and indicate challenges or awareness issues among mothers to ensure their children’s adequate and balanced nutrition. General food insecurity, non-availability or lack of affordability of nutrient-rich foods, time poverty for non-engagement of fathers in child-feeding, and mothers’ engagement in daily wage labour are other known barriers complicating the scenario in similar settings (3, 4, 7, 12–14).

The study was conducted in a Hindu-majority population. Among respondents, more than one-third were young (<25 years old) and marginalized, more than 20% were illiterate, and only about 15% were working. Complementary feeding practices by mothers of children aged 6–11 months and 12–23 months varied significantly across different socio-demographic strata. A lower level of CF practices among marginalized communities called for culturally appropriate targeted intervention to ensure better acceptance. The lag in CF practices in these communities is often related to poorer affordability, lack of access to correct counselling on CF practices from healthcare providers of FLWs, resultant misperceptions, and gaps in knowledge/awareness about the nutritional requirements of a child (3, 4, 7, 12–14, 27, 28).

The education of mothers was found to be a strong driver of better child feeding practices, intuitively because it is crucial to understand the need and implement the recommended practices correctly, as also suggested in several studies (7, 12, 13, 29). Thus, tailoring of interventions to ensure better translation into practice needs to be designed in a manner so that they are easy to understand and follow, for the mothers of infants, especially in settings with educational barriers. Mothers from wealthier households demonstrated better feeding practices, suggesting that economic constraints may be limiting access to appropriate guidance for child nutrition and feeding, as well as resources to address food insecurity, corroborating the findings from Corsi et al. (12) Interestingly, while working mothers, potentially due to better mobility and economic independence, practiced better MDD and MAD, they lacked in TICF, very likely due to their time poverty. In corroboration with our findings, a qualitative study conducted in Wardha district of India outlined reasons such as time constraints and lack of awareness for early initiation, and inadequate complementary feeding among working mothers (14).

Timely introduction of complementary foods is critical during the transition from exclusive breastfeeding to solid foods. Our data shows lower adherence to recommended CF practices among younger infants, suggesting delays in initiation. Mothers often misperceive the needs of infants, fearing indigestion or diarrhoea, while others underestimate meal frequency and quality. TICF, MMF, MDD, and MAD improved among 9–11 months old children and got even better among 12–23 months old. These may be attributed to parents’ perception of increasing dietary need for children with advancing age, expected behavior of introducing a wider range of solid foods with more frequency and quantity to their older children, sharing their meals with older infants, their previous experiences, and accumulated awareness. AMQ remains very less even for the older children, potentially because of misperception about the adequacy of quantity. Wealth status seemed to have affected the CF practices significantly. Practices remaining worse in uneducated, poorer, and marginalized families highlight the negative role of material poverty, food insecurity, lack of access to information, resources, or support systems during that critical period to make informed decisions.

Exposure to programmatic interventions through community platforms/events (participation in the CF-day in the last 3 months), although having a very low coverage (about one third participating), was found to be an effective driver of the IYCF. Traditional and cultural barriers play significant roles in reducing the effectiveness of nutritional interventions owing to a lack of acceptability. To prevent this, inherent heuristics, predilection, and biases embedded deeply within communities are to be well-understood and factored into the design of the interventions. Through programmatic convergence, integration with community platforms (CF-Days, VHSNDs: Village Health Sanitation and Nutrition Days) is needed, with a strong focus on the recommended practices. Intuitive counselling aligned with popular rituals emerged as a potential solution for overcoming long-standing barriers of misperception and wrong IYCF practice. A cluster-randomized controlled trial also highlighted the significant impact of CF behavior change communication delivered through community-level actors on the nutritional outcomes of children (28). Those who received Balshakti from AWC had better CF practices, most likely facilitated by higher coverage of FLW visits and counselling on CF, ensuring utilization. In addition, this direct support with guidance from a health worker is expected to have a positive impact, considering the high acceptance (more than 90% receiving it) and the appropriateness to address the need for at least the poorer sections of society.

Beyond demonstrating statistical associations, the observed relationships point to plausible behavioral and structural mechanisms influencing complementary feeding practices. The contrasting effects of maternal employment are negatively associated with the timely initiation of complementary feeding yet positively associated with dietary diversity and minimum acceptable diet, likely reflecting competing influences of time poverty and economic empowerment. While working mothers may face constraints in initiating age-appropriate feeding due to work demands and caregiving substitution, increased financial autonomy and exposure to information networks may enable greater food diversity and quality, a pattern also reported in previous studies from India and other LMIC settings (12, 14). Similarly, the better feeding practices observed among non-Hindu households may reflect contextual differences in dietary norms, food taboos, and intra-household decision-making, rather than religious affiliation per se, underscoring the need for culturally sensitive and non-homogeneous programmatic approaches.

Programmatic exposures, including participation in CF-day, receipt of Balshakti, and sustained frontline worker engagement, were consistently associated with improved feeding practices, suggesting that repeated interpersonal contact, demonstration-based counselling, and tangible nutritional support play a reinforcing role in translating knowledge into practice. These findings align with evidence indicating that behavior change interventions are most effective when counselling is frequent, context-specific, and embedded within trusted community platforms (17, 28). Collectively, these results highlight that improvements in IYCF practices are driven not only by awareness but also by structural enablers such as time, resources, cultural acceptability, and continuity of support, with important implications for the design of scalable, equity-oriented nutrition interventions.

The notable impact of FLW visits for 6 months post-delivery on the CF practices of mothers also highlights the significance of these at-home interactions, which may have led to discussions on the child’s health and nutrition-related aspects, and hands-on support leading to better CF practices. Counselling on CF in general and specifically on MMF, TICF, AMQ, MDD, and MAD had positive associations with CF practices, and thus highlight the need for targeted and individualized support to mothers in implementing recommended feeding practices. More frequent and higher-quality counselling is also critical for better CF practices among mothers. Major concerns emerged from the very low coverage of AMQ counselling by FLWs and the very low practice of providing AMQ. Minimal counselling on this aspect probably limits mothers’ knowledge of the provision of AMQ to their children, which is also demonstrated in our associational analysis, where counselling on AMQ positively correlated with the practice.

Over the years, various government initiatives continued to seek to improve the nutritional scenario in the country, including the ICDS, the National Health Mission, the Janani Suraksha Yojana, the Matritva Sahyog Yojana, the Mid-Day Meal Scheme, and the National Food Security Mission, among others. In addition to these schemes, the National Nutrition Strategy was released in 2017, targeting the reduction of all forms of malnutrition by 2030, with special focus on the most vulnerable and critical age groups. Our data identifies practice-poor socio-demographic pockets in the community and specific community-level programmatic services that can be targeted to improve community IYCF practices through various systemic approaches. Targeted interventions on awareness and support alongside systemic endeavours in the communities can substantially improve IYCF practices in the targeted districts in Gujarat through the Behavior change process. Previous research also suggests that interventions coupled with addressing the underlying barriers, like material and time poverty, poor education, misconceptions, disease burden, and lack of women’s empowerment, can effectively help to eliminate undernutrition sustainably (17). In addition, interventions aimed at improving health and nutritional awareness, along with access to community support services, may actually correct the dietary practices for infants and young children, especially among marginalized, uneducated, and poor. Engaging with local and national authorities is critical to implement systemic interventions on the ground, with a major focus on the community pockets that are in dire need of tailored attention.

The current study had some important limitations. Like any cross-sectional study, temporal ambiguity was a possibility in our analysis, although we accounted for this through consultation of records. As the sampling frame was established based on existing AWCs, the study might have missed out on some under-resourced areas without AWCs. The self-reported nature of the study is subject to self-selection, social desirability, and recall bias. Self-selection may culminate in some threat to generalizability, and thus, any extrapolation beyond the study sample may be done with caution. Potential for over-reporting of positive behaviors may result from social desirability and differential recall, coupled with it may result in overestimation of the associations. We tried to address these through stringent training and quality control on sampling, ice-breaking, and pre-interview context-setting to reduce drop-out/non-response/misinformation. The data was collected between August and September 2021, which may have coincided with the impacts of the COVID-19 pandemic, ultimately affecting the maternal behaviors and access to health services, and thus affecting the nutritional practices of the mothers. Finally, like any other observational study, the determined associations may not be interpreted as causal.

Despite these limitations, the current study, by virtue of its co-designing with the governmental counterparts, large representative sample, and robust analytics, could generate important insights, which were possible to be disseminated with the government stakeholders in real time. Owing to the buy-in generated based on the data validation, quality assurance, co-creation, and co-facilitation, the governmental system could integrate the findings of the study directly into its program management and decision-making. Findings also helped in designing further intervention (Project Vruddhi) with more ground-truthing and targeted focus.

### Limitations of the study

This study has several limitations. The cross-sectional design precludes causal inference between exposures and infant and young child feeding (IYCF) practices. Data were based on self-reported maternal interviews and may be affected by recall and social desirability bias, particularly for feeding behaviors and counselling exposure. Some stratified and multivariable analyses were affected by sparse data, especially for age-specific or low-prevalence programmatic variables, resulting in small cell sizes and occasional model non-convergence, which limited inference for certain subgroups. Barriers to optimal IYCF practices were not directly measured using validated behavioral or psychosocial tools and were instead inferred from observed associations. Data collection during the COVID-19 pandemic may have additionally influenced service access and feeding practices.

## Conclusion

IYCF practices and access to relevant quality services continued to lag in specific communities in targeted districts of Gujarat. Timely initiation, quantity, frequency, and diversity of CF were found to have community-specific challenges, quite amenable to solving sustainably through targeted interventions. While the reach of interventions through the FLW channel is quite satisfactory for all practices (sans quantity) along with distribution of special take-home ration (Balshakti), the quality of counselling needs to improve, as well as community participation in community-based events like “Annaprasan Diwas.” These programmatic exposures, along with socio-demographic factors like caste, education, time, and material poverty, act as key modifiers of the IYCF practices of the mothers of children below 2 years. Social behavior changes communication strategy with targeted interventions through established channels (FLWs, community-based events/rituals, self-help groups) are critical. These need to be coupled with personalized support to address misperceptions, awareness gaps, and socio-cultural disbeliefs to improve CF towards better nutritional outcomes for these children in the study area. Interventions need to have a nutritional focus integrated functionally with the overall infant and child-health programs, and implement measures and reforms to improve maternal care and nutrition simultaneously. Unless a convergent program focuses on addressing the mother–child dyad as a single target with family as the platform of intervention, the nutritional improvement will always remain an unfinished agenda. Tailored support and clear communication will certainly help mothers and families in better understanding the nutritional requirements of their children and thus, promote healthier feeding practices.

## Data Availability

The original contributions presented in the study are included in the article/supplementary material, further inquiries can be directed to the corresponding author.
